# The first non-mammalian CXCR5 in a teleost fish: molecular cloning and expression analysis in grass carp (*Ctenopharyngodon idella*)

**DOI:** 10.1186/1471-2172-11-25

**Published:** 2010-05-26

**Authors:** Qiao Q Xu, Ming X Chang, Rong H Sun, Fan S Xiao, P Nie

**Affiliations:** 1 State Key Laboratory of Freshwater Ecology and Biotechnology, Institute of Hydrobiology, Chinese Academy of Sciences, Wuhan, Hubei Province 430072, PR China; 2 School of Animal Science, Yangtze University, Jingzhou, Hubei Province 434025, PR China

## Abstract

**Background:**

Chemokines, a group of small and structurally related proteins, mediate chemotaxis of various cell types via chemokine receptors. In mammals, seven different CXC chemokine receptors denoted as CXCR1 to CXCR7 have been reported. However, the chemokine receptor CXCR5 has not been reported in other vertebrates.

**Results:**

In the present study, the genomic sequence of CXCR5 was isolated from the grass carp *Ctenopharyngodon idella*. The cDNA sequence of grass carp CXCR5 (gcCXCR5) consists of 1518 bp with a 43 bp 5' untranslated region (UTR) and a 332 bp 3' UTR, with an open reading frame of 1143 bp encoding 381 amino acids which are predicted to have seven transmembrane helices. The characteristic residues (DRYLAIVHA) and conserved cysteine residues are located in the extracellular regions and in the third to seventh transmembrane domains. The deduced amino acid sequence shows 37.6-66.6% identities with CXCR5 of mammals, avian and other fish species. The grass carp gene consists of two exons, with one intervening intron, spaced over 2081 bp of genomic sequence. Phylogenetic analysis clearly demonstrated that the gcCXCR5 is clustered with those in other teleost fish and then in chicken and mammals. Real-time PCR analysis showed that gcCXCR5 was expressed in all tested organs/tissues and its expression level was the highest in trunk kidney, followed by in the spleen. The expression of gcCXCR5 was significantly modulated by immunostimulants such as peptidoglycan (PGN), lipopolysaccharide (LPS), polyinosinic-polycytidylic acid sodium salt (Poly I:C) and phytohaemagglutinin (PHA).

**Conclusion:**

The cDNA and genomic sequences of CXCR5 have been successfully characterized in a teleost fish, the grass carp. The CXCR5 has in general a constitutive expression in organs/tissues examined, whereas its expression was significantly up-regulated in immune organs and down-regulated in brain, indicating its potential role in immune response and central nervous system.

## Background

Chemokines are a family of small (8-14 kDa), inducible, structurally related proteins, which mediate chemotaxis of various cell types including neutrophils, monocytes, lymphocytes, basophils, eosinophils and fibroblasts to sites of inflammation [1] and are implicated in many biological processes, such as migration of leukocytes, embryogenesis, angiogenesis, hematopoiesis etc [2-5]. The biological activities of chemokines are mediated via chemokine receptors, which belong to a large family of rhodopsin-like G-protein-coupled, seven transmembrane domain receptors [6,7]. Chemokines and their receptors were divided into four families (CXC, CC, C, and CX3C) on the basis of cysteine residues in the ligands (here C represents cysteine and X/X3 represents one or three no-cysteine amino acids) [1]. Recently, a system of nomenclature was introduced in which each ligand and receptor is identified by its subfamily with an identifying number [8]. Thus, there exist CCR1-11, CXCR1-7, XCR1 (the lymphotactin receptor), and CX3CR1 (the fractalkine receptor) [9].

In human and mouse, seven different CXC chemokine receptors denoted as CXCR1 to CXCR7 have so far been reported, and these CXC chemokine receptors have roles in chemotaxis of neutrophils, attraction of Th1 cells, or effector of T cell generation [10]. Genomic structure and expression of five CXCRs including CXCR1, 2, 3, 4, 7 have been characterized either in model fish and/or in economically important fish species. For example, CXCR1 has been reported in several species of fish, such as common carp *Cyprinus carpio *and mandarin fish *Siniperca chuatsi *[11,12]; CXCR2 in common carp [12]; CXCR3 in grass carp *Ctenopharyngodon idella *[13]; CXCR4 in sea lamprey *Petromyzon marinus*, zebrafish *Danio rerio*, common carp, rainbow trout *Oncorhynchus mykiss*, sterlet *Acipenser ruthenus *[14-18], CXCR7 in zebrafish and medaka *Oryzias latipes *[19,20]. In teleost fish, the literature on the function of CXC chemokine receptors is rather limited. It was only recently reported that two CXCR4 genes, CXCR4a and CXCR4b, isolated in zebrafish had roles in the development and migration of cranial neural crest cells [21]. Similar to CXCR4, it was demonstrated that CXCR7, which was recently revealed to recognize the chemokine, stromal cell-derived factor-1 (SDF1) [22], played an essential role in primordium migration [23], and CXCR4 and CXCR7 are antagonistic in control of cell migration in the development of the posterior lateral line [23].

CXCR5 was first reported from human Burkitt's lymphoma [24], whereafter the murine homologue of CXCR5 was cloned and its expression was found in a pattern similar to human CXCR5 [25]. In mammals, CXCR5 and its ligand CXCL13 are responsible for the organization of B cell follicles and the migration of B and T cells [26,27], and involved in other functions such as in the attraction of human metastatic neuroblastoma cells to the bone marrow [28]. However, CXCR5 has not been identified in any species of fish so far. In this study, CXCR5 was cloned from the grass carp *C. idella*, an important fish in aquaculture industry of China [29]. Furthermore, its expression was examined in different organs/tissues, and in response to the stimulation of peptidoglycan (PGN), lipopolysaccharide (LPS), polyinosinic-polytidylic acid sodium salt (Poly I:C) and phytohaemagglutinin (PHA).

## Results

### Cloning and characterization of grass carp CXCR5 cDNA

**Figure 1:**
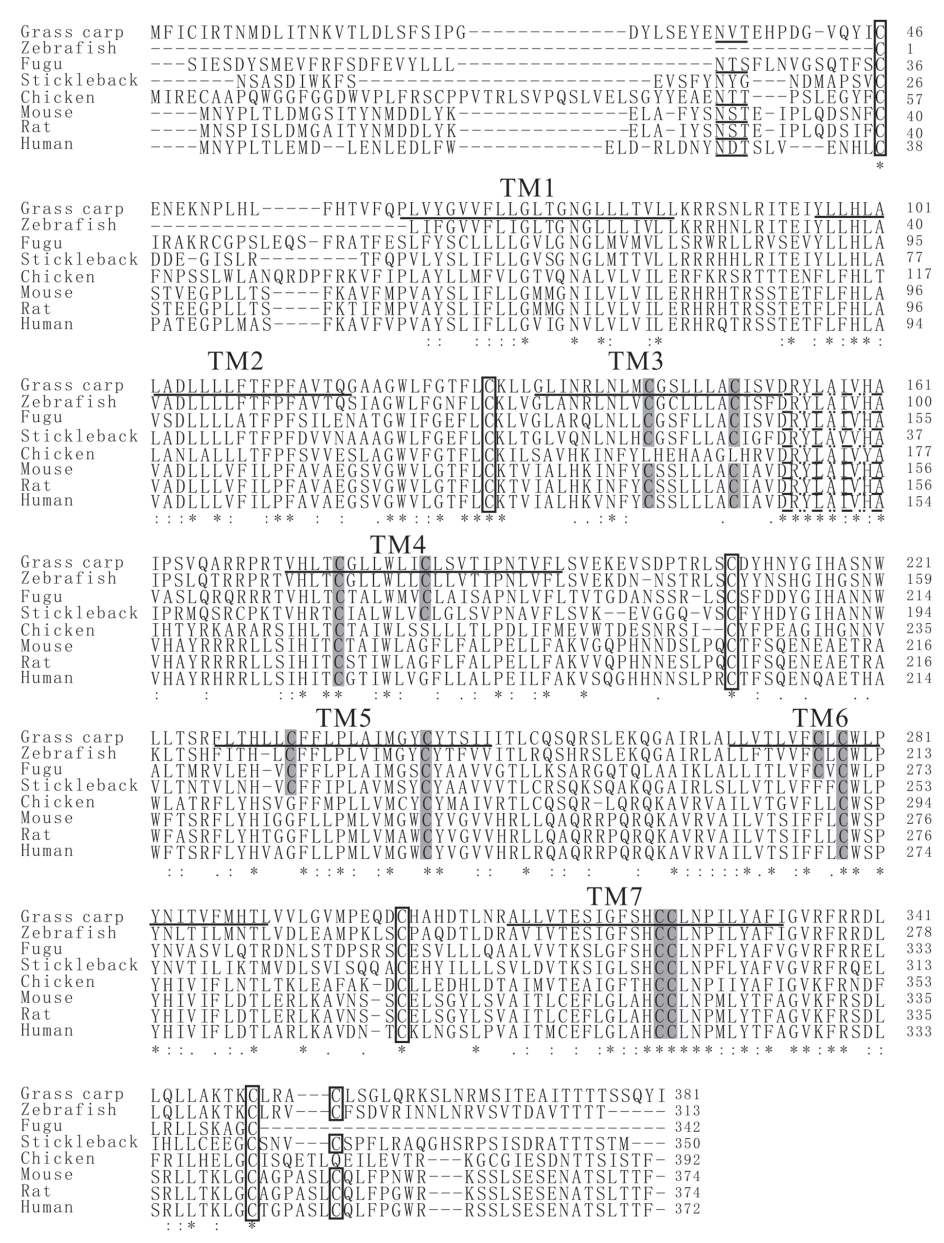
**The alignment of the amino acid sequences of gcCXCR5 (GenBank accession no. **FJ825363**) with CXCR5 sequences of other vertebrates by using CLUSTAL W program**. Symbol (*) represents identical residues, (:) conservative substitution, (.) similar residues and (-) missing residues. The seven transmembrane domains are underlined, cysteines in the transmembrane domains are indicated by gray shadow and the conserved four cysteines presumably forming disulfide bonds are boxed. Broken underline indicates sequence DRYLAIVHA and uninterrupted underline NXS/T indicates N-glycosylation site. The sequences of CXCR5 used for analysis are derived from the GenBank, with their accession numbers listed in Table 2.

The grass carp CXCR5 (gcCXCR5) cDNA (GenBank accession no. FJ825363) consists of 1648 bp with a 43 bp 5' untranslated region (UTR) and a 332 bp 3'-UTR with poly(A) addition signal (AATAAA) and two mRNA instability motifs (ATTTA). The open reading frame of gcCXCR5 cDNA encodes 381 amino acids with a calculated molecular mass of 43.34 kDa and an isoelectric point of 8.40. Analysis of the putative amino acid sequence by TMpred program suggests that gcCXCR5 is a membrane protein with seven transmembrane helices between amino acids 65-87, 99-121, 136-155, 176-198, 230-252, 272-294 and 314-336 (Figure 1). The extracellular regions of the gcCXCR5 contain four cysteines, presumably forming disulfide bonds. In the transmembrane regions TM3-TM7 existed respectively two other cysteine residues. Moreover, there are two cysteine intracellular regions at the carbon terminal. Analysis using Signal P-N program showed that gcCXCR5 has no signal peptide, but has a potential N-glycosylation sites at N34. Using the Scanprosite programs in PROSITE database http://ca.expasy.org/prosite, a sequence CGSLLLACISVDRYLAI (145-161 amino acids) containing the G-protein-coupled receptor family 1 signature was identified. It was also revealed that the gcCXCR5 sequence contains cAMP- and cGMP- dependent protein kinase phosphorylation sites (at amino acids 85-88), tyrosine kinase phosphorylation site (at amino acids 206-214), N-myristoylation sites (at amino acids 72-82, 125-130 and 215-220), protein kinase C phosphorylation sites (at amino acids 13-15, 224-226 and 255-257), and casein kinase II phosphorylation sites (at amino acids 7-10, 30-33 and 367-370) (Figure 1).

**Figure 2:**
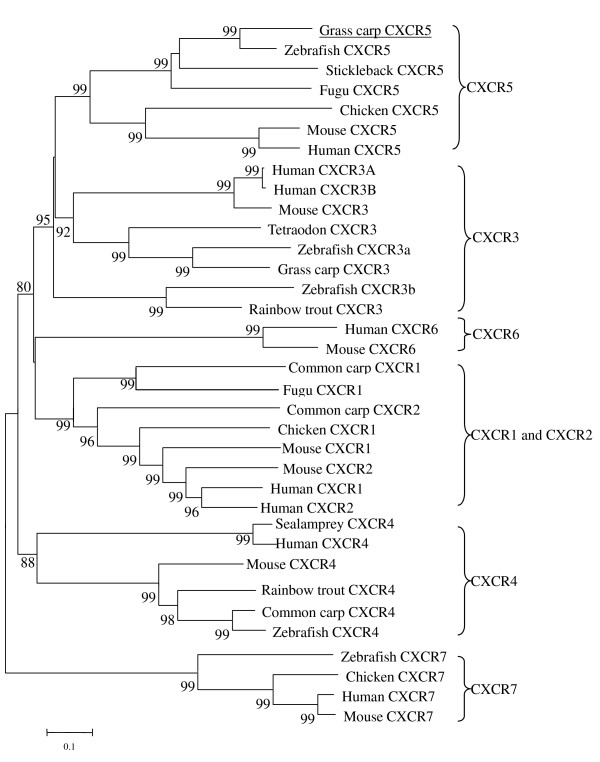
**Phylogenetic relationship of CXCR members**. A neighbor-joining phylogenetic tree of chemokine receptor sequences was constructed based on analysis of protein sequences by the computer program CLUSTER W. The sequences of the CXCR used for analysis are derived from the GenBank and SwissProt, and their accession numbers are listed in Table 2. The bootstrap confidence values shown at the nodes of the tree are based on 1000 bootstrap replications. *Homo sapiens*, Human; *Mus musculus*, Mouse; *Gallus gallus*, Chicken; *Danio rerio*, Zebrafish; *Cyprinus carpio*, Common carp; *Ctenopharyngodon idella*, Grass carp; *Oncorhynchus mykiss*, Rainbow trout; *Petromyzon marinu*s, Sea lamprey; *Tetraodon nigroviridis*, Tetraodon; *Takifugu rubripes*, Fugu; *Gasterosteus aculeatus*, Stickleback.

The deduced amino acid sequence of the gcCXCR5 protein was compared with other chemokine receptors by calculating the sequence identities using the MEGALIGN program. The full-length amino acid sequence of gcCXCR5 has the highest identities with zebrafish CXCR5 (66.6%), followed by stickleback CXCR5 (53.5%) and fugu CXCR5 (51.6%). The identifies of gcCXCR5 with CXCR5 from human, mouse, rat and chicken are 38.8%, 37.8%, 37.6% and 37.6%, respectively. The identity between gcCXCR5 and other reported chemokine receptors, CXCR1, CXCR2, CXCR3, CXCR4, CXCR6 and CXCR7, ranges among 27.3-33.2%, 31.2-34.0%, 35.0-43.5%, 27.8-30.4%, 26.4-28.0%, and 36.5-37.1%, respectively. In the phylogenetic tree based on amino-acid sequences from four CXCR1, three CXCR2, eight CXCR3, six CXCR4, seven CXCR5, two CXCR6 and four CXCR7 sequences, the gcCXCR5 was clustered closely (bootstrap value > 99%) with CXCR5 in other fish species, and then with other CXCR5 in higher vertebrates (Figure 2), and all other CXCRs with same identified number were clustered into a same clade, except that CXCR1 and CXCR2 were clustered together.

### Characterization of gcCXCR5 genomic DNA

**Figure 3:**
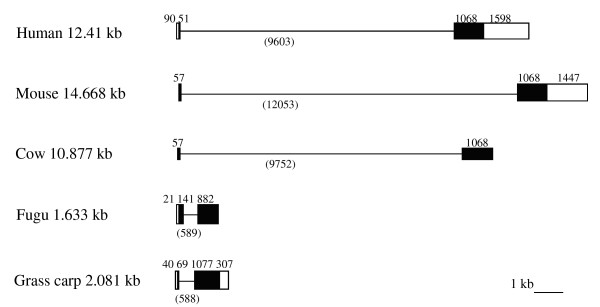
**Schematic diagram of exon-intron arrangement of CXCR5 genes from human (Gene ID 643), mouse (Gene ID 12145), cow (Gene ID 497021), fugu (ENSTRUG00000011814) and grass carp (GenBank accession no. **FJ825364**)**. Boxes represent exons and lines adjacent to exons represent introns. Open reading frames and untranslated regions are shown as black boxes and white boxes, respectively. The number of nucleotides in each exon and intron is, respectively, shown above and below the corresponding element.

The gcCXCR5 gene has a length of 2081 bp (GenBank accession no. FJ825364). Using BLAST2, the alignment of gcCXCR5 cDNA sequence with gcCXCR5 genomic sequence revealed that the gcCXCR5 gene is composed of two exons and one intron. The first exon is 109 bp in length, containing a short 5'-UTR and ORF of 23 amino acids (Figure 3). The second exon is 1384 bp in length, coding for the all seven transmembrane domains of gcCXCR5. The intervening intron is 588 bp. The exon-intron junction follows the consensus rule of the splice donor and acceptor sites for splicing (Figures 1 and 3).

### Expression of gcCXCR5

**Figure 4:**
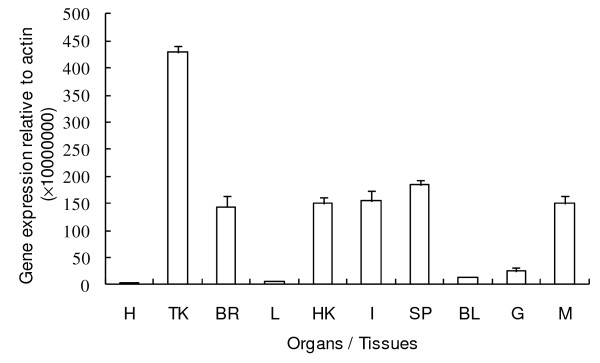
**Expression of gcCXCR5 in different organs/tissues revealed by real-time PCR**. Total RNA was obtained from heart (H), trunk kidney (TK), brain (BR), liver (L), head kidney (HK), intestine (I), spleen (SP), blood (BL), gill (G) and muscle (M), respectively, then were reverse-transcribed to cDNA. Gene expression was measured by means of real-time quantitative PCR and is shown relative to the gene expression of β-actin.

In normal fish, the expression of gcCXCR5 was observed in all the organs/tissues examined, with strong expression in trunk kidney, spleen, intestine, head kidney, brain, muscle, but almost undetectable expression in liver and heart (Figure 4).

**Figure 5:**
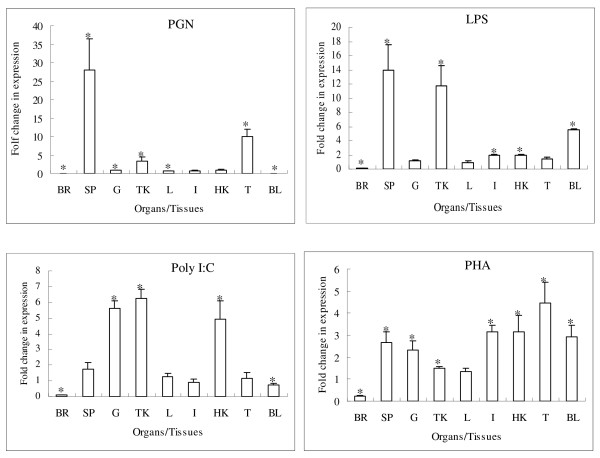
**Expression pattern of grass carp CXCR5 gene induced by peptidoglycan (PGN), lipopolysaccharide (LPS), polyinosinic-polycytidylic acid sodium salt (Poly I:C) and phytohaemagglutinin (PHA) in vivo**. Total RNA was obtained from brain (BR), spleen (SP), gill (G), trunk kidney (TK), liver (L), intestine (I), head kidney (HK), thymus (T) and blood (BL), respectively. The transcriptional changes are shown in ratio in comparison with control fish, as determined by quantitative real-time PCR. Each column and bar represents the mean ± SE of three individual fish.

As shown in Figure 5, PGN injection significantly induced gcCXCR5 expression in spleen, thymus and trunk kidney, decreased expression in brain, gill, liver and blood (*P *< 0.05), but no significant difference in other organs/tissues (*P *> 0.05). LPS stimulation significantly increased gcCXCR5 expression in spleen, trunk kidney, blood, intestine and head kidney (*P *< 0.05), but decreased expression in brain (*P *< 0.05), with no significant difference in other organs/tissues (*P *> 0.05). The induced expression of gcCXCR5 by Poly I:C was observed in trunk kidney, head kidney and gill (*P *< 0.05), decreased expression in brain and blood (*P *< 0.05). PHA significantly induced gcCXCR5 in most organs/tissues (*P *< 0.05) except brain and liver. The decreased expression of gcCXCR5 by PHA was observed in brain (*P *< 0.05).

## Discussion

CXCR5, also known as BLR1, has been identified as a member of the lymphocyte-specific GPCR family [30]. The present study for the first time reported the cDNA and genomic sequences of CXCR5 and its expression pattern in a teleost fish, the grass carp. In general, the sequences of chemokine receptors have 25-80% aa identity. However, many other G-protein-coupled peptide receptors (GPCRs) also have around 25% aa identity with chemokine receptors, suggesting that the structural boundary is not very sharp. Although they lack a single structural signature, there are several features that together are found more frequently among chemokine receptors than in other types of GPCRs. These include a length of 340-370 aa, an acidic N-terminal segment, the sequence DRYLAIVHA or a variation of it in the second intracellular loop, a short basic third intracellular loop, and a cysteine in each of the four extracellular domains. In addition, chemokine receptors contain numerous serines and threonines in the C-terminal tail that become phosphorylated after receptor-ligand interaction [8,31]. The sequence of gcCXCR5 contains all these characters except for the length of amino acid sequences. The alignment of gcCXCR5 amino acid sequence with CXCR5 sequences from other vertebrates revealed some conserved structural features. The presence of N-linked glycosylation sites at N-terminus and/or in the second extracellular loop is a common feature of chemokine receptors, as recognized by other authors [7,13,32], and all the CXCR5s including gcCXCR5 contain a conserved N-linked glycosylation sites at N terminus. Furthermore, the close phylogenetic relationship between gcCXCR5 and zebrafish CXCR5, and then other teleost fish CXCR5 may indicate their close evolutionary relationship. This, together with other CXCR members, i.e., CXCR3, CXCR4, CXCR5, CXCR6, which were clustered respectively into different clades, may reveal to some extent the conservation of these members in vertebrates, except that CXCR1 and CXCR2 were clustered in a same clade, as shown also by other reports [22,33], which may imply a similar evolutionary origin of these two receptors.

Similar to the genomic structures of CXCR5 in fugu (ENSTRUG00000011814), human (Gene ID 643), mouse (Gene ID 12145) and cow (Gene ID 497021), the gcCXCR5 consists of two exons and one intron. Compared with the size of mammalian CXCR5 genomic sequences, fish CXCR5 is much smaller in length. Despite the difference in genomic size, vertebrate CXCR5 have similar size in exons.

In mammals, much effort has been focused on identifying CXCR5 expression in different organs/tissues and also in cell types [25,30]. Murine homologue of CXCR5 has been described as being expressed in lymphoid organs, and murine CXCR5-specific RNA is detected consistently at low levels in secondary lymphatic organs. The CXCR5 gene is expressed regularly and strongly in lymphomas of mature B cells but not in plasmacytomas [25,30]. In the present study, constitutive expression of CXCR5 was observed abundantly in trunk kidney, spleen, head kidney, intestine, muscle and brain. In vivo, grass carp CXCR5 expression was up-regulated mainly in immune organs such as spleen, trunk kidney, head kidney and blood, suggesting the potential function of gcCXCR5 in immune response. The induced expression of gcCXCR5 in a wider range of organs/tissues containing lymphocytes was consistent with the function of mammalian CXCR5, as reported to attract T lymphocytes [26,27]. The immunostimulants used in the present study include PGN, LPS and Poly I:C, which are derived from Gram-positive bacteria, Gram-negative bacterial endotoxin and a synthetic double stranded RNA (dsRNA) mimicking viral dsRNA, respectively; and phytohaemagglutinin (PHA), known to cause leucocyte agglutination and to stimulate the proliferation of lymphocytes [34]. The modulated difference of gcCXCR5 by different immunostimulants may be owing to the effect of different pattern recognition receptors (PRRs) which recognize different bacterial and/or viral elements on different cell types.

On the other hand, it is difficult to explain the down-regulation of gcCXCR5 expression in brain after PGN, LPS, Poly I:C and PHA stimulation. However, the higher expression of gcCXCR5 in brain was similar to gcCXCR3 which also had abundant expression in brain [13]. Based on the observation in mammals that CXCR2, CXCR3 and CXCR4 were expressed in central nervous system by neurons and microglial cells etc [31,35-37], it is suggested that brain chemokine receptors may promote the recruitment of haematopoietic cells from circulation, both as part of normal surveillance and immunological control within the brain, and as a component of the inflammatory response [38]. Whether this is the case for fish CXCR5 and other fish CXCRs requires further study.

In addition, CXCR5 may be modulated by other cytogenes. Krumbholz et al. [39] reported that about 20% of CSFCD4^+ ^cells and almost all B cells expressed the CXCL13 receptor CXCR5. In vitro, CXCL13 was produced by monocytes and at a much higher level by macrophages. CXCL13 mRNA and protein expression was induced by TNF alpha and IL-1beta but inhibited by IL-4 and IFN gamma [39]. However, it would be interesting to know if this is the case in fish.

## Conclusions

In summary, gcCXCR5 consists of 1518 bp, encoding 381 amino acids which are predicted to have seven transmembrane helices. The characteristic residues (DRYLAIVHA) and conserved cysteine residues are located predominantly in the extracellular regions and in the third to seventh transmembrane domains. The gcCXCR5 was expressed in all tested organs/tissues. The expression of gcCXCR5 was significantly modulated by peptidoglycan, lipopolysaccharide, polyinosinic-polycytidylic acid sodium salt and phytohaemagglutinin.

## Methods

### Cloning of cDNA and genomic sequences

Based on the zebrafish and Tetraodon sequences of CXCR5 homologues from Ensembl website, one pair of degenerate primers F1 and R1 were designed to obtain the internal region of gcCXCR5. The PCR cycling conditions were 1 cycle of 94°C for 5 min, 35 cycles of 94°C for 30 s, 57°C for 30 s and 72°C for 30 s, followed by 1 cycle of 72°C for 10 min. The resultant product was isolated using the Gel Extraction Kit (Omega, USA), cloned into pMD18-T vector (TaKaRa, Japan) and transformed into *Escherichia coli *strain M15 competent cells by following the manufacturer's instruction. Putative clones were screened by PCR using the above primers under the same PCR cycle conditions, and the selected clones were sequenced. To obtain the full-length cDNA sequence of gcCXCR5, 5' RACE and 3' RACE were performed by using the gene-specific primers and adaptor primers. The universal primers mix (UPM) was the mixture of the long form (UPM Long) and short form (UPM Short).

For the first 3'-RACE, the PCR was initially performed with primers UPM/3-F1 followed by a nested PCR with primers UPM/3-F2. The annealing temperature of first and second PCR was 63°C and 65°C, respectively. For the second 3'-RACE, the PCR was performed with primers UPM/3-F3 followed by a nested PCR with primers UPM/3-F4. The annealing temperature of first and second PCR was 63°C and 66°C, respectively.

For 5'-RACE, first strand cDNA synthesis is primed using a gene-specific antisense primer (5-R1). Following cDNA synthesis, the first strand product is purified from unincorporated dNTPs and 5-R1. TdT (Terminal deoxynucleotidyl transferase) is used to add homopolymeric tails to the 3' ends of the cDNA. Tailed cDNA is then amplified by PCR using gene-specific antisense primer (5-R2) and 5' abridged anchor primer (AAP). The annealing temperature of PCR was 55°C. A dilution of the original PCR (0.1%) was re-amplified using a gene-specific antisense nested primer (5-R3) and abridged universal amplification primer (AUAP). The annealing temperature of PCR was 65°C. All primers are listed in Table 1.

**Table 1:** Oligonucleotide primers used to amplify the gcCXCR5 gene

Name	Sequence (5'-3')	Usage
F1	ATCTACCTGCTA(G)CAC(T)CTGGC	Cloning for the internal fragment
R1	AGCAGC(G)TGC(G)AGCAGGTCG(T)C(T)T	

UPM Long	CTAATACGACTCACTATAGGGCAAGCAGTGGTATCAACGCAGAGT	Race-PCR Universal primers
UPM Short	CTAATACGACTCACTATAGGGC	

AAP	GGCCACGCGTCGACTAGTACGGGIIGGGIIGGGIIG	5' RACE Abridged Primer
AUAP	GGCCACGCGTCGACTAGTAC	

5-R1	GGTTCCAAAAAGCCA	5'RACE 1^st ^round PCR
5-R2	CTTGAGTAACTGCGAATGGAAA	5'RACE 2^nd ^round PCR
5-R3	GAGCAGGTCAGCCAGTGCCAGAT	5'RACE 3^rd ^round PCR

3-F1	GTTTTCTGCCTGTGCTGGCTACCG	3'RACE 1^st ^round PCR
3-F2	CCCTGGTAGTGTTGGGTGTTATGCC	3'RACE 2^nd ^round PCR

3-F3	CCGTTTCCGAAGAGACCTCCTGC	3'RACE 1^st ^round PCR
3-F4	GCCATTACTACCACAACAAGCAGC	3'RACE 2^nd ^round PCR

D-F1	TGGACGAGGACACACACTTCTGAG	For intron
D-R1	GCCGTTTTAGCAGGACTGTCAAGAG	

β-actinF	CCTTCTTGGGTATGGAGTCTTGAGAGTATTTACGCTCAGGTGGG	Real-time quantitative PCR control
β-actinR		

RT-F1	GTGTTGGGTGTTATGCCTGAGATGTATTGGCTGCTTGTTGTG	Real-time quantitative PCR
RT-R1		

The genomic DNA was purified from trunk kidney of healthy grass carp using Wizard Genomic DNA Purification Kit (Promega). Based on the full-length cDNA sequence, D-F1 and D-R1 were designed to obtain the full-length genomic sequence of gcCXCR5. PCR was performed using the primer pairs listed in Table 1.

### Sequence analysis

Protein prediction was performed using software at the ExPASy Molecular Biology Server http://www.expasy.ch/. The putative ORFs were analyzed for the presence of signal peptides using the algorithms Signal P 3.0. The intron/exon structure of the identified genomic sequence was determined by alignment of the full-length cDNA to the genomic sequence using BLAST2 http://www.ncbi.nlm.nih.gov/blast/bl2seq/wblast2.cgi. The transmembrane regions were identified by the Tmpred http://www.cbs.dtu.dk/services/TMHMM/. Putative domains and the G-protein-coupled receptors family 1 signature were identified by PROSITE http://ca.expasy.org/prosite. A multiple alignment was generated using the CLUSTAL W program. Sequence identities were calculated using the MEGALIGN program within DNASTAR. Phylogenetic analysis was performed using the neighbor-joining method within the Mega molecular evolutionary genetic analysis software package. Data were analyzed using Poisson correction, and gaps were removed by pairwise deletion. The degree of confidence for each branch point was determined by bootstrap analysis (1,000 times). All the sequences used for the phylogenetic analysis are listed in Table 2.

**Table 2:** The accession numbers of chemokine receptor sequences used for phylogenetic tree construction and multiple sequence alignment

Species	Gene	Accession no.	Species	Gene	Accession no.
*Homo sapiens*	CXCR1	NP_000625	*Gallus gallus*	CXCR1	NP_001026762

	CXCR2	NM_001557		CXCR5	NP_001026083

	CXCR3A	P49682		CXCR7	NM_001083362

	CXCR3B	NP_001136269	*Danio rerio*	CXCR3a	NP_001082899

	CXCR4	NP_001008540		CXCR3b	NP_001007315

	CXCR5	NP_001707		CXCR4	NP_571909

	CXCR6	NP_006555		CXCR5	XP_002665447

	CXCR7	NP_064707		CXCR7	NM_001083832

*Mus musculus*	CXCR1	NP_839972	*Cyprinus carpio*	CXCR1	BAA31458

	CXCR2	NM_009909		CXCR2	BAA31470

	CXCR3	NP_034040		CXCR4	BAA32797

	CXCR4	NP_034041	*Ctenopharyngodon idella*	CXCR3	AAW69766

	CXCR5	NP_031577		CXCR5	FJ825363

	CXCR6	NP_109637	*Oncorhynchus mykiss*	CXCR3	CAC86390

	CXCR7	NP_031748		CXCR4	NP_001117814

*Rattus norvegicus*	CXCR5	NP_445755	*Takifugu rubripes*	CXCR1	NP_001072110

*Tetraodon nigroviridis*	CXCR3	CAF98051		CXCR5	ENSTRUG00000011814

*Petromyzon marinus*	CXCR4	AAO21209	*Gasterosteus aculeatus*	CXCR5	ENSGACG00000012278

### RNA extraction and cDNA synthesis for expression analysis

Grass carp, 200 to 300 g in body weight, were obtained in Niushan lake, Wuhan, Hubei Province, China. After seven-day acclimatization in a quarantine tank, heart, trunk kidney, brain, liver, head kidney, intestine, spleen, blood, gill and muscle from three grass carp were used for RNA isolation using Trizol reagent (Invitrogen, USA) in order to analyze the expression of gcCXCR5 in healthy grass carp.

To study the effect of different immunostimulants on the expression of gcCXCR5, four stimulants with different origins and different functions were chosen. Phytohaemagglutinin (PHA) is an extract from plant with roles in stimulating lymphocyte proliferation, lipopolysaccharide (LPS) and peptidoglycan (PGN) were derived from Gram- negative and positive bacteria, respectively, while Poly I:C mimics virus. Five groups of fish (three fish each) were injected with either 500 μl PBS, 500 μl PGN (1 mg/ml), 500 μl PHA (1 mg/ml), 500 μl Poly I:C (2 mg/ml), or 500 μl LPS (2 mg/ml). Twenty-four hours after injection, total RNA was extracted using TRIzol reagent (Gibco) as described by the manufacturer from organs/tissues of interest, including brain, spleen, gill, trunk kidney, liver, intestine, head kidney, thymus and blood from both injected and control groups.

After treatment with RNase-free DNase I, 2 μg of total RNA was reverse-transcribed respectively with Revert Aid TM First Strand cDNA Synthesis Kit (Fermentas). All cDNA samples were stored at -20°C until used in real-time PCR assays.

### Real-time quantitative PCR

Primer premier 5.0 was used for designing forward and reverse primers. The primers which performed best in real-time PCR were: gcCXCR5 Forward (RT-F1), Reverse (RT-R1); β-actin Forward (β-actin F), Reverse (β-actin R) (Table 1). The annealing temperatures for gcCXCR5 and β-actin were 58°C, and resultant amplicons of both were 266 and 221 bp, respectively. The gcCXCR5 and β-actin cDNA fragments were generated by PCR. Amplicons were gel-purified, cloned into pMD18-T vector and transformed into *Escherichia coli *strain DH5α competent cells. Cloned amplicon sequences were confirmed by sequencing. Plasmid DNA was obtained by using the Plasmid mini kit I (Omega) by following the manufacturer's instructions. Serial tenfold dilutions of plasmid DNA were used in PCR for establishing a standard curve. PCR reactions were performed using Chromo 4™ Continuous Fluorescence Detector from MJ Research. Amplifications were carried out at a final volume of 20 μl containing 1 μl DNA sample, 10 μl 2 × SYBR green Real time PCR Master Mix (Toyobo, Japan), 2 μl of each primer and 5 μl H_2_O. PCR amplification consisted of 5 min at 95°C, followed by 45 cycles consisting of 10 s at 94°C, 15 s at 58°C, 20 s at 72°C and plate-reading at 80°C. The reaction carried out without DNA sample was used as control. Melting curve analysis of amplification products was performed at the end of each PCR reaction to confirm that a single PCR product was detected. Each sample was run in triplicate. Standard curves were run on the same plate.

Statistical analysis was performed using a one-way analysis of variance (ANOVA). A probability level of *P *< 0.05 was considered significant. Fold change was calculated as (Ts/Tn)/(Cs/Cn) where Ts equals the treated sample assayed for the specific gene and Tn equals the treated sample assayed for β-actin gene, and Cs and Cn equal the calibrator group with the specific and normalizing gene, respectively [40]. All statistical analyses were based on comparisons between the control and injection groups.

## Authors' contributions

XQQ carried out the experiments and drafted the manuscript. CMX participated in the study design and in the manuscript preparation. SRH cloned partial sequence. XFS was involved in the experiment. NP was responsible for overall project and finalized the manuscript. All authors read and approved the final manuscript.
